# Establishing the Replicability and Generalizability of Multi-Study Longitudinal Research

**DOI:** 10.1093/geroni/igaa057.1879

**Published:** 2020-12-16

**Authors:** Scott Hofer

**Affiliations:** University of Victoria, Victoria, British Columbia, Canada

## Abstract

Replication and cross-validation of research findings across independent longitudinal studies is essential for a cumulative science. However, the interplay between harmonization, replication, and generalizability of results across interdisciplinary longitudinal studies can present remarkable challenges. Careful interpretation of multistudy results must include consideration of the age, birth cohort, health, and education of individuals in the sample, measurements, the number and spacing of assessments, and rates of response and attrition. Placed in a broader historical (or future) context, we must consider the representativeness of population sampling, historical period, and analytic method in understanding the replicability and generalizability of findings. In a multistudy context, harmonization can be considered at levels of research question, statistical models, and measurements and can minimize some sources of cross-study variability. I will discuss the challenges and benefits of harmonization and the coordinated analysis approach used by the IALSA research network to achieve results from multi-study integrative research.

